# Mandatory Labelling of CMR Substances in Implants and Their Impact on Orthopedics and Trauma Surgery

**DOI:** 10.1055/a-2761-5480

**Published:** 2026-02-13

**Authors:** Jan Birkholz, Jonas Roos, Hannes Hasselbach, Reinhold Preißler, Marc D. Michel, Dieter Christian Wirtz, Michael M. Morlock

**Affiliations:** 1 Quality Management 72196 Peter Brehm GmbHWeisendorfDeutschland; 2 Klinik und Poliklinik für Orthopädie und Unfallchirurgie 39062 Universitätsklinikum BonnBonnNRWDeutschland; 3 MedizinrechtPreißler Ohlmann & Partner mbB RechtsanwälteFürthDeutschland; 4 Geschäftsführung 72196 Peter Brehm GmbHWeisendorfBayernDeutschland; 5 Institut für Biomechanik 38987 TUHHHamburgHamburgDeutschland

**Keywords:** hazardous substances, labeling, implant, CMR, cobalt

## Abstract

With the commencement of EU Regulation 2017/745 (Medical Device Regulation, MDR), a justification and labelling obligation for medical devices was introduced for substances (chemicals, elements) that are proven or suspected to be carcinogenic, mutagenic or toxic to reproduction (CMR substances). This also applies to substances having endocrine disrupting properties. This obligation has led to great concern in the industry and among medical professionals, particularly with regard to possible uncertainty on the part of patients and users. The aim of this article is to clarify the most important context in order to counteract these concerns. For patients, the CMR labelling requirement will not result in any changes in the quality of care. On the other hand, the medical profession has the additional task of conveying a sense of security to their patients despite an uncontrollable public debate. The use of a ceramic head instead of a metal head in hip joint replacement can be cited as an example here. CMR-labelled implants pose only a very low risk to the patient due to the material, which only occurs in very rare cases if the restoration fails. In the future, however, manufacturers must aim to qualify more CMR-free materials. However, this is a cost- and time-intensive endeavour under the regulations of the MDR with high requirements, particularly with regard to clinical efficacy. The well-established tried-and-tested implant materials have proven to be clinically successful even in difficult revision situations. Switching completely to a different material without the corresponding clinical experience could potentially lead to losses in component safety. The implementation of the CMR labelling requirement should be closely monitored by both the medical profession and manufacturers in order to counteract any uncertainty among patients at an early stage.

## Introduction

With the entry into force on 26 May 2021, EU Regulation 2017/745 (Medical Device Regulation, MDR) introduced a justification and labelling obligation for medical devices that contain substances (chemicals, elements) whose effect as carcinogenic, mutagenic, or reprotoxic (CMR substances) is proven or suspected 1 . This also applies to substances with endocrine-disrupting properties. This move has led to significant concern in industry and the medical profession, especially with regard to possible uncertainty on the part of patients and users. This paper aims to clarify the most important relationships to counteract this uncertainty.

## Regulatory Background

### About the REACH and CLP regulations on CMR labelling

A wide range of materials and chemicals are used in the manufacture of medical devices. The devices generally pass multiple successive processing and trade stages until they reach the end user for their intended use.

For many years, there was no systematic information on most chemicals, especially those already on the European market before 1981. Manufacturers of these substances were only obliged to provide missing information when a substance assessment by the authorities showed information gaps or there were indications of a risk to the environment or health. This process proved slow and cumbersome. For example, the use of asbestos in the construction industry or various substances in pesticides from agriculture was only regulated once the risks to humans and the environment became known.

Against this background, a comprehensive overhaul of EU chemicals legislation entered into force in 2007 with the introduction of Regulation (EC) 1907/2006 2 . This regulation requires, prior to placing chemicals and substances on the market, a

- Registration,
- Evaluation and
- Authorisation

of Chemicals (REACH Regulation).

The REACH Regulation also introduced the concept of “substances of very high concern” and led to the establishment of the “European Chemicals Agency” (ECHA) based in Helsinki, Finland. “Substances of concern” include CMR substances. These are substances proven or suspected to be

- Carcinogenic,
- Mutagenic, or
- Reprotoxic

With the introduction of the REACH regulation, their market access was subject to new restrictive and comprehensive rules. No chemical may be marketed without registration by the manufacturer and an associated regulatory review by the ECHA. At the same time, manufacturers must assess the risk potential of the substances themselves and prove their safe use. This is done by means of “safety data sheets”, which provide information on the potential danger posed by certain chemicals or substances.

The REACH Regulation requires that information on the safe handling of hazardous chemicals be shared through every stage of the supply chain. This chain of information must not be broken, from the original manufacturer through processing and intermediaries to the end user. The REACH Regulation therefore also places obligations on importers and (end) users of chemicals.

In 2008, just one year after the Reach regulation was introduced, the CLP Regulation on the Classification, Labelling, and Packaging of substances and mixtures (1272/2008) was adopted as a supplement to the REACH Regulation 3 .

This Regulation classifies CMR substances into different hazard classes and, based on existing evidence, classifies them into the following subcategories 4 :

- 1A (proven CMR effect in humans),
- 1B (suspected CMR effect based on animal studies) or
- 2 (substances suspected of being CMR based on limited human or animal studies)

With the date of application of EU Regulation 2017/745, known as the Medical Device Regulation (MDR), a justification and labelling obligation for medical devices was established as of 26 May 2021 for both CMR substances and substances with endocrine-disrupting properties, which have also been incorporated into the REACH Regulation 5 . This now makes it legally binding that medical devices may contain CMR substances or endocrines in concentrations above 0.1% by weight only if this can be justified, but without further specifying this justification. It is therefore the responsibility of the manufacturer to justify the choice of the CMR substance. There is no specific guidance from the MDR on how this justification should be presented structurally and substantively, or which criteria should be applied.

Irrespective of the specific effect, CMR substances and/or endocrines are in any case subject to labelling if

- a substance is classified as 1A or 1B according to the CLP Regulation (e.g. cobalt); and
- the (material) has direct patient contact (e.g. surgically invasive implants and instruments).

The manufacturer of a medical device must mark the presence of CMR materials directly on the device and/or its packaging, and must also mark them in the instructions for use (IFU) and patient labels (e.g. for the implant identification card). The legislature even stipulates that each CMR substance must be individually labelled 5 .

For implantable devices, the information in the IFU must contain all qualitative and quantitative information on the materials and substances with which patients come into contact 6 . In addition to identifying the chemical CMR substance itself, the specific amount of the substance in question must be indicated.

In certain cases, information on residual risks and, if applicable, appropriate precautionary measures is legally required in addition to the basic labelling obligation. Such information is necessary in particular when using devices whose intended purpose is

- the treatment of children; or
- the treatment of pregnant or breast-feeding women; or
- treatment of other patient groups considered particularly vulnerable to such substances and/or materials 7 .

For the labelling of CMR substances, symbols may be used which are applied in a similar way to a pictogram (e.g. on the label). If symbols from uniform standards and regulations – known as harmonised standards 8 – are used, the presence of CMR or endocrine substances may be indicated on the label of the medical device by means of the appropriate symbol, with further lists and explanations provided in the instructions for use 9 .

## Current and Future Practices Regarding the Labelling Requirements of Implants

Alongside synthetic materials and ceramics, metallic materials are predominantly used in orthopaedics and traumatology. In hip endoprosthetics, the most common of these is titanium in cement-free implants, including in the form of high-strength titanium alloys (e.g. Ti6Al4V). Cemented hip and knee prostheses predominantly contain cobalt chrome (e.g. Co28Cr6Mo) and/or stainless steel alloys (e.g. FeCrNiMnMoNbN). Titanium alloys, ceramics and polyethylene, do not contain any CMR substances that must be labelled.

However, cobalt, as the main component of the base alloy of cobalt-chrome alloys with a concentration of 55–70% by weight, is the primary CMR substance in orthopaedics and trauma surgery and will have to be labelled in the future.

Due to the raw materials, steel alloys inherently contain certain proportions of tramp elements, sometimes also as permissible impurities, which are not regulated by the standards or for which only an upper limit is set. Cobalt is frequently also present in these tramp elements and can amount to > 0.1% by weight. For this reason, surgical instruments used for implantation, which are mostly made of stainless steel, will in future also be subject to labelling 10 .

For several decades, almost all of the materials listed have proven their worth in everyday clinical practice, and their chemical compositions and the required material properties are regulated in numerous internationally harmonised standards. The series of standards of ISO 5832 “Implants for surgery – Metallic materials” serves as an example. This standard originated in the 1990 s and has been regularly updated since then.

## Labelling Obligations of the Medical Device Manufacturer

**Fig. 1:**
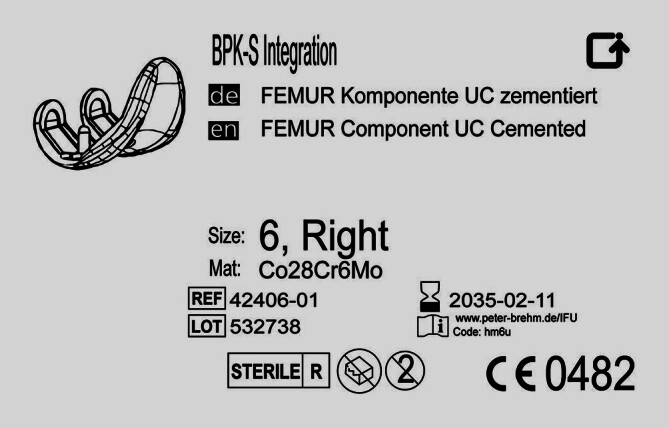
Current label for a knee implant (femoral component), showing the use of standardised symbols in accordance with DIN EN ISO 15223-1:2019. Reproduced with permission from DIN – Deutsches Institut für Normung e.V., Berlin.

**Fig. 2:**
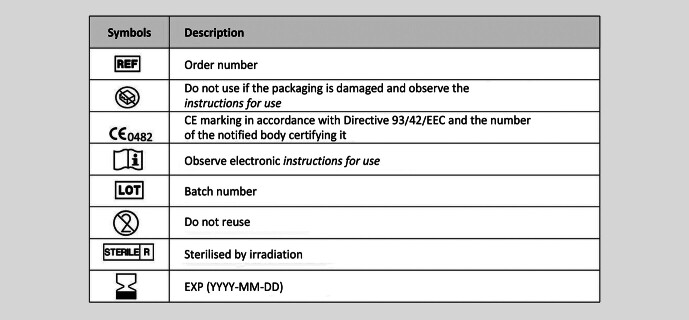
Explanation of the symbols used in Figure 1 in accordance with DIN EN ISO 15223-1:2019. Reproduced with permission from DIN – Deutsches Institut für Normung e.V., Berlin.

**Fig. 3:**
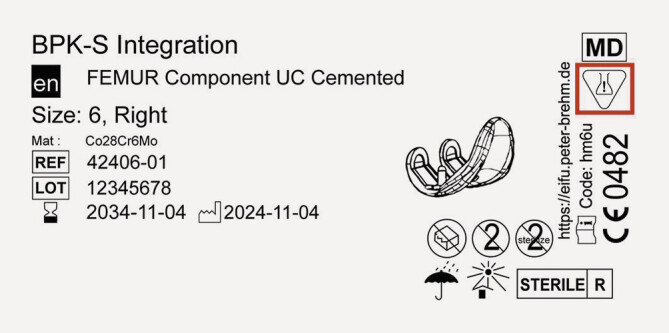
This label meets the requirements of the MDR for the same knee implant as shown in Figure 1 and uses standardised symbols in accordance with DIN EN ISO 15223-1:2021. Reproduced with permission from DIN – Deutsches Institut für Normung e.V., Berlin.

**Fig. 4:**
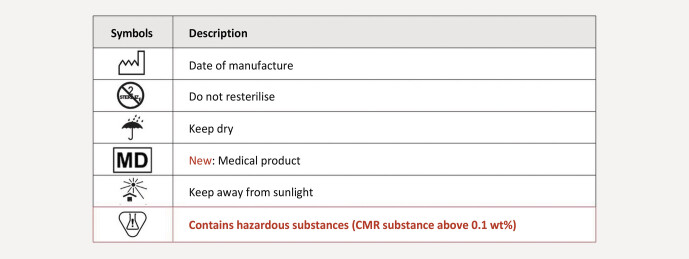
Explanation of the symbols used in Figure 3 in accordance with DIN EN ISO 15223-1:2021. Reproduced with permission from DIN – Deutsches Institut für Normung e.V., Berlin.

Currently, manufacturers of endoprostheses explicitly state the materials used on the label. The label for a knee prosthesis (femur component) is used here by way of example and follows the specifications and regulations valid to date (see Fig. 1 and the associated explanations in Fig. 2 ). The date of application of the MDR with the introduction of the limit value of 0.1% by weight for the labelling obligation of the individual CMR substances now obliges manufacturers to extend previous information on the labels at least by the normatively defined symbol for hazardous substances (see Fig. 3 and the associated explanations in Fig. 4 ).

Surgical instruments should be treated analogously: Here, too, the legislator stipulates that the instruments must be marked directly on the product (e.g. by laser marking). The symbol for identifying CMR substances is also mandatory.

## Consequences of the CMR Labelling Requirement for Manufacturers of Medical Devices in Orthopaedics and Trauma Surgery

On its homepage, the European Chemical Agency (ECHA) has a summary of all chemical substances that comply with the requirements of the MDR, CLP and REACH 11 .

As of 6 March 2024, a total of 6959 substances is listed. Of these, 2592 are potentially subject to the justification and labelling obligation for medical technology manufacturers (some substances may fall into more than one classification category):

- 353 carcinogenic 1A
- 856 carcinogenic 1B
- 433 mutagenic 1B
- 655 reprotoxic 1A
- 295 reprotoxic 1B

This list is updated by the ECHA every 3 to 6 months. Following an update, MedTech manufacturers are obliged to extend the labels with a transitional period of 18 months, if their respective products are affected. Manufacturers are expected to face significant and rapid changes concerning substances subject to labelling requirements.

Implant materials used in large joint replacements have not undergone any serious material changes over the last few decades, so the workload involved in the updates remains basically manageable. MDR-related re-certification requires that even unchanged and long-established and successful existing products must also be updated to comply with the new labelling requirements.

Explaining to patients that a CMR label does not necessarily indicate a risk will be challenging.

In addition to the mandatory implant identification card and the information provided to date, patients will in future also be informed of the presence of CMR substances. To what extent patients can adequately assess these warnings without prior knowledge, or whether this information obligation instead leads to considerable hesitation, refusal, or anxiety regarding the implant, must be closely monitored. Further product information, such as the instructions for use, is not generally available to patients.

## Medical Implications of CMR Labelling Obligations and Their Impact on Present and Future Patient Management

From a medical point of view, any physician treating patients with endoprosthetics should ensure that they are informed of the reasons for CMR labelling, the underlying scientific principles and the regulatory requirements. This is generally the case when using CMR-marked implants, but especially in implants whose design may result in metal debris.

Metal-on-metal (MoM) articulations became widespread in the early 2000 s but are now rarely used. The current share of MoM articulations in hip endoprosthetics in Germany is about 0.1% 12 . Their limited use today is the increased release of metal ions caused by abrasion or corrosion observed under unfavourable conditions 13 . While there is currently no reliable evidence that the implants have carcinogenic or teratogenic effects, the available data are not sufficient to completely exclude such potential effects. The concentration of metal ions can be measured in urine or blood and elevated levels which may indicate local or systemic metallosis can be detected 14 . However, there are no mandatory limit values 15 .

For example, a known problem is pseudotumor, which occurs in about 1% of patients within the first 5 years 16 . In addition, contact allergy to metals can be an issue in endoprosthetic care with a reported incidence of 20.4% for nickel, 3.4% for cobalt and 1.5% for chrome 17 . For these cases, hypoallergenic prosthesis variants are used, which can be coated in different ways (titanium-niobium nitride). However, compared to standard prostheses, no significant difference was found in the 5-year comparison for cobalt concentration in blood plasma in primary knee endoprosthetics despite the technical advantage 18 . Since allergy patients frequently suffer from anxiety disorders, there is a risk that the labelling of hazardous substances will further worsen the outcome 19 .

As a precautionary measure, EFORT (European Federation of National Associations of Orthopaedics and Traumatology) and MedTech Europe’s Orthopaedic Sector Group issued a joint statement on 14 August 2018stressing that the labelling requirement, irrespective of whether a CMR substance is a real risk, could potentially mislead the health community. It was therefore recommended that clarification be provided that, while the substance itself is classified as hazardous, any risks resulting from its presence in the device have been duly assessed, minimised and managed 20 . In addition, the Association of the German Dental Industry e. V. (Verband der Deutschen Dental-Industrie e. V. - VDDI) issued a statement on cobalt in dental alloys, concluding that cobalt-based alloys remain a valuable and currently irreplaceable therapeutic agent in dentistry 21 . Indeed, CMR labelling can lead to general uncertainty, as it is not currently routinely mentioned in patient-doctor discussions and patients could only become aware of it by, for instance, labelling in their implant identification card ( Fig. 2 ). Even in the majority of patients with implanted MoM articulations, it is unlikely that short- to medium-term complications associated with their constituent CMR substances will arise 22 .

In order to avoid unjustified patient concerns, it is highly recommended from a medical perspective that this issue be addressed transparently and informatively. This could be done in the form of information materials produced by the manufacturer in digital and/or print versions, so that patients clear and easily understandable information about the CMR marking and its implications.

## Implications of the CMR Labelling Requirement from an Engineering Perspective and Future Supply

The new labelling requirements are intended to inform the user that a particular medical device (such as an implant) contains a CMR substance, but also to confirm that the producer has managed all the risks caused by this substance. Therefore, the labelling as such is not a cause for concern with regard to the safety of orthopaedic implants.

The concentration of the CMR substance within the alloy of the implant is the decisive factor that determines whether the patient will encounter problems. Only in cases of severe implant-related problems can the concentration increase to such an extent that it poses a risk to the patient’s health. This issue was significantly intensified by the introduction of large-head metal hip replacement prostheses, as critically high cobalt and chromium concentrations with clinical consequences occurred more frequently in patients with this type of prosthesis. In the absence of this type of prosthesis and of patients who implanted with a metal head following a ceramic head fracture 23 24 , the risk of mortality, cardiovascular disease, cancer and neurodegenerative disorders is not increased in patients with cobalt–chromium-containing hip prostheses 25 .

In knee arthroplasty, metal debris is an extremely rare cause of failure of a total knee replacement and has essentially only been reported in cases of abnormal metal-on-metal contact or severe third-body wear 26 . Allergy-related issues play a frequently discussed role in knee replacement; however, the scientific assessment is highly heterogeneous, and there is no direct connection to the issue of CMR.

The topic of cobalt is discussed in detail by way of example. An assessment of the scientific literature clearly shows that, although a potential risk exists, cobalt alloys – due to their unique combination of properties such as strength, durability, and a long history of safe use – are particularly well suited for use in a wide range of medical devices 27 .

Evaluations of the relevant preclinical and clinical data on the carcinogenicity and reprotoxicity of cobalt alloys, conducted to meet the requirements of the EU Medical Devices Regulation, support the conclusion that exposure to cobalt alloys in medical devices via clinically relevant routes does not pose a risk of carcinogenicity or reprotoxicity 27 28 29 . Furthermore, the risk of adverse effects known to occur at elevated cobalt concentrations (e.g. cardiomyopathy) is very low (rare reports, often reflecting one single catastrophic failure event among millions of patients) 24 .

In summary, the favourable benefit-risk profile, even compared with possible alternative materials, supports the continued use of cobalt alloys in medical devices.

The classification of the metal cobalt by the European Commission as a Category 1B carcinogen (presumed to have carcinogenic potential) was based primarily on data from rodent inhalation carcinogenicity studies 23 .

The author group conducted a systematic review and a meta-analysis to assess the risks of specific cancer types associated with cobalt exposure through total joint replacement (TJR) or occupational exposure (OC). Results were stratified by exposure type (OC or TJR), exposure level (metal-on-metal (MoM) or non-MoM), follow-up duration (latency period: < 5, 5–10 or > 10 years), and cancer incidence or mortality (detection bias assessment). From 30 studies (653,104 subjects, average 14.5 years follow-up), the association between TJR/OC and cancer risk was null for 22 of 27 cancer sites, negative for 3 sites, and positive for prostate cancer and myeloma. Significant heterogeneity and large estimate ranges were observed for many cancer sites. No significant increase in estimates was observed by exposure level or follow-up duration.

The current evidence, including weak associations, heterogeneity across studies and no increased association with exposure level or follow-up duration, is insufficient to conclude that there exists an increased risk for people exposed to cobalt in TJR/OC of developing site-specific cancers.

Numerous studies have come to a similar conclusion. In summary, meta-analyses did not show any relationship between occupational exposure to orthopaedic implants containing cobalt alloys or cobalt particles and overall cancer risk, including an analysis of studies directly comparing metal-on-metal implants with non-metal-on-metal implants 23 24 25 26 29 .

## Consequences of the CMR Labelling Requirement from a Legal Perspective

### General

Medical treatment must comply with the generally recognised standards of medical care applying at the time of treatment (known as the specialist standard), unless otherwise agreed, cf. § 630a (2) of the German Civil Code (BGB). The obligation applies directly to the contracting party of the treatment agreement, such as the practice owner or the hospital operator, and indirectly to the treating physician, who is not a contracting party 30 .

### Treatment errors

If the treatment does not meet the generally accepted professional standards, this constitutes a violation of the obligations under the treatment contract in the form of malpractice. This includes the use of a defective medical device, the medically unacceptable selection or misuse of a medical device or the violation of other safety-related organisational obligations 31 .

The CMR marking does not impair the marketability of CE-marked medical devices nor does it indicate that they are faulty. Patient health protection is adequately covered by the required CE marking 32 , so that the use of CMR-marked medical devices per se cannot be malpractice. A different situation can only arise if there is positive scientific evidence of increased health risks resulting from the use of CMR-labelled products.

### Informed consent

Prior to implementing medical treatment, in particular an intervention into the body or health, the treating party is obliged to obtain consent from the patient, cf. § 630d (1) sentence 1 of the German Civil Code (BGB). The validity of consent requires appropriate (so-called risk or self-determination) information, cf. § 630d (2) in conjunction with § 630e of the German Civil Code (BGB).

The content of the information includes, among other things, the anticipated consequences and risks involved in the measure, cf. § 630e (1) sentence 2 of the German Civil Code (BGB). Therefore, the patient must also be informed about the risks arising from the use of medical devices 33 . This must be provided orally, cf. § 630e (2) sentence 1 no. 1 of the German Civil Code (BGB), which is why a reference to the manufacturer’s instructions alone is not sufficient 31 .

For some time, it has been known that in prostheses with metal-on-metal articulations, the risk of metal debris may occur. This can lead, among other things, to cobalt and chromium deposition in the human body 34 . According to the prevailing view, patients should be informed about these risks 35 . Insofar as the potential health hazards of metal debris can be attributed to the CMR properties of the materials used, it is at least advisable to provide information on these specific properties and any associated risks.

There is no requirement to provide information on purely theoretical risks for which there is neither practical experience nor confirmation in studies 36 . This should also apply if the instructions for use include information on theoretical risks that do not need to be explained to the patient 31 .

Against this background, for example in the case of the risk of metal debris in endoprostheses, a distinction can be made: Metal debris does not constitute a theoretical risk; like the associated and measurable metal deposits in the human body, it represents a real risk 37 , and therefore must be explained accordingly to the patient. The same can be assumed for the generally known health-damaging properties of the corresponding materials, in particular of CMR substances. A different approach may apply, however, to specific health impairments feared as a result of CMR substance deposition, insofar as no experience with these has yet been reported in the medical community. In this case, the risks would be purely theoretical and therefore not subject to the obligation to provide information 38 .

In addition to the risk or self-determination information, according to § 630e (1) sentence 3 German Civil Code, patients must also be informed of treatment alternatives. This is the case when several medically equally indicated and common treatments can lead to significantly different demands, risks or chances of cure 39 . According to the legal literature and case law, the choice of material for endoprostheses is not an alternative treatment which requires clarification, provided that, according to medical expertise at the time of treatment, the selected material does not pose an increased health risk 40 .

### Consequences of CMR labelling of medical devices in the context of the treatment contract

In that regard, it is apparent that the obligation to label CMR active substances under medical device law has no direct effect on the obligations under the treatment contract. If there are actual health risks for the patient arising from CMR substances contained in the implants – for example in rare cases due to metal debris – the patient must be informed of these risks within the framework of risk disclosure and informed consent.

## Consequences of the CMR Labelling Requirement from the Perspective of Patients – Patients who Already Have Implants and Those Who are to Receive Implants in the Future

The new CMR labelling requirement for medical devices can lead to uncertainty for patients, both those with already implanted prostheses and those planning an implantation in the future. However, there is no increased risk arising from products that have already been implanted simply because they have been retrospectively assigned a CMR label. The CMR label only reflects regulatory requirements and does not indicate any change in the risk profile of devices already implanted. However, there is a risk of unnecessary uncertainty and fear of complications when patients learn about the CMR classification of their implants only after they have been implanted. This that this classification is purely regulatory must be communicated clearly to avoid such fears and to maintain trust. Patients should be told that the safety and efficacy of their implants remain unchanged.

With regard to future treatment, that the labelling likewise does not generally entail an increased risk. The new labelling requirements do not, in any way, imply a decline in existing treatment or in the quality or safety of the implants. The new labelling requirements do not imply any technical changes to the products and therefore no change to clinical practice and the quality of care. Rather, they serve solely to ensure transparency and patient protection in the rare cases in which material-related problems with implants may arise.

Therefore, the CMR label does not indicate a treatment error when implants bearing this label are used. The duty to inform continues to apply only when risks are demonstrably known in the medical literature in connection with the use of an implant, such as in the case of metal-on-metal articulations using cobalt-chromium alloys. If an implant is known to pose increased health risks, it is advisable to inform the patient about possible alternatives.

Both previously treated patients and new patients should be told that the implants used in endoprosthetic care are safe and have been tried and tested for years.

## Summary Recommendations – What’s in Store? Outlook

For patients, the CMR labelling requirement will not result in any changes in the quality of care. Medical professionals, however, now have the added responsibility of reassuring their patients, despite an uncontrollable public debate. Here, the use of a ceramic head instead of a metal head in hip joint replacement can be cited as an example.

As stated above, CMR-labelled implants only bear a very low material-based risk for the patient, which arises only in very rare instances of treatment failure. Looking ahead, manufacturers must, however, aim to qualify more CMR-free materials. Yet under the regulations of the MDR, this is a costly and time-consuming undertaking, particularly due to the stringent requirements for clinical efficacy.

The well-established implant materials have also proven to be clinically successful in challenging revision situations. A complete switch to another material without the appropriate clinical experience could potentially lead to a loss of component safety.

Both physicians and manufacturers should actively implement the CMR labelling requirement to counteract patient uncertainty from the outset.

## Notes

Extra Info The labelling obligations apply universally, irrespective of whether the (implant) material represents a concrete hazard to patients.
