# Supplementary material for: Mandatory Labelling of CMR Substances in Implants and Their Impact on Orthopedics and Trauma Surgery
Source: Z Orthop Unfall. 2026 Feb 13;164(3):265–73. doi: 10.1055/a-2761-5480 (PMC13285750; doi:10.1055/a-2761-5480)
Supplement: Related texts

# Review article: Kennzeichnungspflicht von CMR-Stoffen bei Implantaten und deren Impact auf Orthopädie und Unfallchirurgie

**Keywords:** CMR, Kennzeichnung, Implantat, Kobalt

## Abstract

Mit dem Geltungsbeginn der EU-Verordnung 2017/745 (Medical Device Regulation, MDR) wurde zum 26.05.2021 für Stoffe (Chemikalien, Elemente), deren Wirkung als karzinogen, mutagen oder reproduktionstoxisch (CMR-Stoffe) nachgewiesen ist oder vermutet wird, eine Rechtfertigungs- und Kennzeichnungspflicht für Medizinprodukte eingeführt. Dies gilt auch für Stoffe mit endokrin wirkenden Eigenschaften. Dieser Schritt hat zu einer großen Besorgnis in der Industrie und der Ärzteschaft geführt, besonders hinsichtlich möglicher Verunsicherung aufseiten der Patienten und Anwender. Das Ziel dieses Beitrages ist die Klarstellung der wichtigsten Zusammenhänge, um dieser Verunsicherung entgegenzuwirken. Für den Patienten und die Patientin werden sich durch die CMR-Kennzeichnungspflicht keinerlei Veränderungen in der Versorgungsqualität ergeben. Hingegen stellt sich für die Ärzteschaft die zusätzliche Aufgabe, ihren Patienten das Gefühl der Sicherheit trotz einer nicht zu kontrollierenden Diskussion in der Öffentlichkeit zu vermitteln. Hier kann die Verwendung eines Keramikkopfes anstelle eines Metallkopfes beim Hüftgelenkersatz als Beispiel angeführt werden. Von CMR-gekennzeichneten Implantaten geht nur ein sehr geringes Risiko aufgrund des Materials für den Patienten aus, welches nur in sehr seltenen Fällen beim Versagen der Versorgung auftritt. Perspektivisch müssen Hersteller jedoch das Ziel haben, mehr CMR-freie Materialien zu qualifizieren. Allerdings ist dies unter den Regulierungen der MDR mit hohen Anforderungen, insbesondere zur klinischen Wirksamkeit, ein kosten- und zeitintensives Unterfangen. Die bewährten Implantatwerkstoffe haben sich auch bei schwierigen Wechselsituationen als klinisch erfolgreich gezeigt. Der vollumfängliche Umstieg auf ein anderes Material ohne die entsprechende klinische Erfahrung könnte möglicherweise zu Einbußen bei der Bauteilsicherheit führen. Die Umsetzung der CMR-Kennzeichnungspflicht sollte sowohl durch die Ärzteschaft als auch durch die Hersteller intensiv verfolgt werden, um frühzeitig einer Verunsicherung der Patienten entgegenwirken zu können.

## Einführung

Mit dem Geltungsbeginn der EU-Verordnung 2017/745 (Medical Device Regulation, MDR) wurde zum 26.05.2021 für Stoffe (Chemikalien, Elemente), deren Wirkung als karzinogen, mutagen oder reproduktionstoxisch (CMR-Stoffe) nachgewiesen ist oder vermutet wird, eine Rechtfertigungs- und Kennzeichnungspflicht für Medizinprodukte eingeführt 1 . Dies gilt auch für Stoffe mit endokrin wirkenden Eigenschaften. Dieser Schritt hat zu einer großen Besorgnis in der Industrie und der Ärzteschaft geführt, besonders hinsichtlich möglicher Verunsicherung aufseiten der Patienten und Anwender. Das Ziel dieses Beitrages ist die Klarstellung der wichtigsten Zusammenhänge, um dieser Verunsicherung entgegenzuwirken.

## Regulatorischer Hintergrund

## Über die REACH- und CLP-Verordnung zur CMR-Kennzeichnung

Bei der Herstellung von Medizinprodukten kommen vielfältige Materialien und Chemikalien zum Einsatz. Die Produkte durchlaufen i. d. R. mehrere, aufeinander aufbauende Verarbeitungs- und Handelsstufen, bis sie beim Endverbraucher zur bestimmungsgemäßen Verwendung landen.

Über die meisten Chemikalien lagen lange Jahre keine systematisch erhobenen Informationen vor, insbesondere für Chemikalien, die sich bereits vor 1981 auf dem europäischen Markt befanden. Hersteller dieser Stoffe waren erst dann verpflichtet, fehlende Informationen vorzulegen, wenn eine Stoffbewertung der Behörden Informationslücken nachwies oder Hinweise auf eine Gefährdung von Umwelt oder Gesundheit vorlagen. Diese Vorgehensweise stellte sich als langsam und schwerfällig heraus. Beispielsweise wurde die Verwendung von Asbest in der Baubranche oder aber verschiedene Stoffe in Pestiziden aus der Landwirtschaft erst nach Bekanntwerden von Gefährdungen für Menschen und Umwelt im Nachgang reguliert.

Vor diesem Hintergrund trat im Jahr 2007 mit der Verordnung 1907/2006 2 eine umfassende Neuregelung des EU-Chemikalienrechts in Kraft. Diese Verordnung sieht vor dem Einbringen der Chemikalien und Stoffe in den Markt eine

- Registrierung,
- Evaluierung und
- Autorisierung

der Chemikalien vor (REACH Verordnung).

Mit der REACH Verordnung wurde gleichzeitig auch der Begriff der „besonders besorgniserregenden Stoffe“ ins Leben gerufen und eine „Europäische Agentur für chemische Stoffe“ (ECHA) mit Sitz in Helsinki/Finnland gegründet. Unter „besorgniserregende Stoffe“ fallen auch die sog. CMR-Stoffe. Unter dem Begriff CMR werden diejenigen Stoffe zusammengefasst, deren Wirkung als

- karzinogen,
- mutagen oder
- reproduktionstoxisch

nachgewiesen ist oder vermutet wird.

Der Marktzugang wurde damit restriktiv und umfassend neu geregelt. Ohne Registrierung durch den Hersteller mit verbundener behördlicher Prüfung durch die ECHA darf keine Chemikalie vertrieben werden. Zugleich muss der Hersteller das Risikopotenzial der Stoffe selbst bewerten und die sichere Verwendung nachweisen. Dies erfolgt mittels sog. Sicherheitsdatenblätter, die über das von bestimmten Chemikalien oder Stoffen ausgehende Gefahrenpotenzial aufklären.

Die REACH-Verordnung fordert, dass über die gesamte Lieferkette hinweg Informationen zum sicheren Umgang mit den gefährlichen Chemikalien weitergegeben werden. Diese Informationskette darf vom ursprünglichen Hersteller über die Weiterverarbeitung und Zwischenhandel bis hin zum Endkunden nicht abreißen. Somit nimmt die REACH-Verordnung auch Importeure und (End-)Anwender von Chemikalien mit in die Pflicht.

Bereits 1 Jahr später im Jahr 2008 wurde ergänzend zur REACH-Verordnung die CLP-Verordnung zur Einstufung (Classification), Kennzeichnung (Labelling) und Verpackung (Packaging) von Stoffen und Gemischen 1272/2008 3 verabschiedet.

Diese Verordnung stuft CMR-Stoffe in verschiedene Gefahrenklassen und teilt basierend auf den bestehenden Nachweisen in folgende Untergruppen ein 4 :

- 1A (nachgewiesene CMR-Wirkung beim Menschen),
- 1B (vermutete CMR-Wirkung basierend auf Tierstudien) bzw.
- 2 (Stoffe, die basierend auf begrenzten humanen bzw. Tierstudien im Verdacht stehen, CMR zu sein).

Mit dem Geltungsbeginn der EU-Verordnung 2017/745, bekannt als Medical Device Regulation (MDR), wurden zum 26.05.2021 sowohl für die CMR-Stoffe als auch für Stoffe mit endokrin wirkenden Eigenschaften, die ebenfalls in der REACH-Verordnung Eingang gefunden haben, eine Rechtfertigungs- und Kennzeichnungspflicht für Medizinprodukte eingeführt 5 . Hierdurch ist nun gesetzlich verbindlich geregelt, dass CMR-Stoffe oder Endokrine in einem Medizinprodukt nur dann in einer Konzentration von mehr als 0,1% Masseanteil enthalten sein dürfen, wenn dies gerechtfertigt werden kann, ohne dies jedoch genauer zu spezifizieren. Es ist somit Aufgabe des Herstellers, die Wahl des CMR-Stoffes zu rechtfertigen. Eine konkrete Vorgabe, wie diese Rechtfertigung strukturell und inhaltlich darzulegen ist und welche Kriterien angewendet wurden, existiert seitens der MDR nicht.

Unabhängig von der jeweiligen Wirkung sind CMR-Stoffe und/oder Endokrine in jedem Fall kennzeichnungspflichtig, wenn

- ein Stoff als 1A oder 1B gemäß der CLP-Verordnung klassifiziert ist (z. B. Kobalt) und
- der (Werk-)Stoff direkten Patientenkontakt hat (z. B. chirurgisch invasive Implantate und Instrumente).

Der Hersteller eines Medizinproduktes muss das Vorhandensein von CMR-Werkstoffen unmittelbar auf dem Produkt und/oder seiner Verpackung sowie zwingend in den Gebrauchsanweisungen („Instructions for Use“, IFU) und den Patientenetiketten kennzeichnen (z. B. für den Implantatepass). Der Gesetzgeber sieht bei dieser Kennzeichnung sogar vor, dass jeder CMR-Stoff einzeln kennzeichnungspflichtig ist 5 .

Die Angaben in der IFU müssen bei implantierbaren Produkten die gesamten qualitativen und quantitativen Informationen zu den Werkstoffen und Stoffen, mit denen Patienten in Berührung kommen, enthalten 6 . Damit ist neben dem chemischen CMR-Stoff selbst auch die konkrete Menge des jeweiligen Stoffes anzugeben.

In bestimmten Fällen sind über die reine Kennzeichnungspflicht hinaus Informationen zu Restrisiken und ggf. angemessene Vorsichtsmaßnahmen gesetzlich verpflichtend. Solche Informationen sind insbesondere bei der Verwendung von Produkten notwendig, deren Zweckbestimmung

- die Behandlung von Kindern oder
- die Behandlung von schwangeren oder stillenden Frauen oder
- die Behandlung von anderen Patientengruppen, die als besonders anfällig für diese Stoffe und/oder Werkstoffe sind 7 ,

ist.

Für die Kennzeichnung von CMR-Stoffen können Symbole genutzt werden, die ähnlich wie ein Piktogramm aufgebracht werden (z. B. auf dem Etikett). Sofern dabei Symbole aus einheitlichen Standards und Regelwerken – sog. harmonisierten Normen 8 – verwendet werden, besteht die Möglichkeit, durch das entsprechende Symbol auf dem Etikett des Medizinproduktes auf das Vorhandensein von CMR- oder endokrinen Stoffen hinzuweisen und weitere Auflistungen und Erklärungen in der Gebrauchsanweisung anzuführen 9 .

## Aktuelle und zukünftige Praxis der Kennzeichnungspflicht von Implantaten

In Orthopädie und Traumatologie kommen neben Kunststoffen und Keramiken überwiegend metallische Werkstoffe zum Einsatz. Hierzu zählen in der Hüftendoprothetik bei den zementfreien Implantaten überwiegend Titan u. a. als hochfeste Titanlegierungen (z. B. Ti _6_ Al _4_ V). Zementierte Prothesen an Hüfte und Knie bestehen überwiegend aus Kobalt-Chrom- (z. B. Co _28_ Cr _6_ Mo) und/oder Edelstahl-Legierungen (z. B. FeCrNiMnMoNbN). Titanlegierungen, Keramiken und auch Polyethylen, enthalten keine kennzeichnungspflichtigen CMR-Stoffe.

Kobalt jedoch, als Hauptbestandteil der Basislegierung von Kobalt-Chrom-Legierungen mit einer Konzentration von 55–70 Gew.-%, ist der Hauptvertreter der CMR-Stoffe in Orthopädie und Unfallchirurgie und ist zukünftig kennzeichnungspflichtig.

Stahllegierungen enthalten rohstoffbedingt immer gewisse Anteile an Begleitelementen, z. T. auch als zulässige Verunreinigungen, die nicht durch die Normen geregelt sind bzw. für die nur eine Obergrenze festgelegt ist. Bei diesen Begleitelementen ist häufig auch Kobalt vertreten und kann Mengen > 0,1 Gew.-% betragen. Für die für die Implantation genutzten chirurgischen Instrumente, die meist aus rostfreiem Edelstahl bestehen, besteht deshalb in Zukunft ebenso die Kennzeichnungspflicht 10 .

Nahezu alle aufgeführten Materialien haben sich seit mehreren Jahrzehnten millionenfach im klinischen Alltag bewährt und ihre chemischen Zusammensetzungen sowie die erforderlichen Materialeigenschaften sind in zahlreichen international harmonisierten Normen geregelt. Als Beispiel kann hier die Normenreihe der ISO 5832 „Chirurgische Implantate – Metallische Werkstoffe“ dienen, die ihren Ursprung bereits in den 1990er-Jahren hat und seitdem regelmäßig aktualisiert wird.

## Kennzeichnungspflichten des Herstellers des Medizinproduktes

Derzeit geben Hersteller von Endoprothesen auf dem Etikett die verwendeten Materialien explizit an. Als Beispiel dient hier das Etikett für eine Knieprothese (Femur-Komponente) nach den bis dato gültigen Vorgaben und Regelungen (siehe Abb. 1 und den zugehörigen Erläuterungen in Abb. 2 ).Der Geltungsbeginn der MDR mit der Einführung des Grenzwertes von 0,1% Masseanteil für die Kennzeichnungspflicht der einzelnen CMR-Stoffe verpflichtet nun die Hersteller, bisherige Angaben auf den Etiketten mindestens um das normativ definierte Symbol für Gefahrstoffe zu erweitern (siehe Abb. 3 ) und den zugehörigen Erläuterungen in Abb. 4 .

**Abb. 1:** Derzeitiges Etikett für ein Knie-Implantat (Femur-Komponente); Verwendung standardisierter Symbole gemäß DIN EN ISO 15223–1:2019. Reproduziert mit Genehmigung des DIN – Deutsches Institut für Normung e. V., Berlin.

**Abb. 2:** Erläuterung der Symbole aus Abb. 1 ; Verwendung standardisierter Symbole gemäß DIN EN ISO 15223–1:2019. Reproduziert mit Genehmigung des DIN – Deutsches Institut für Normung e. V., Berlin.

**Abb. 3:** Etikett nach MDR-Vorgaben für das gleiche Knie-Implantat wie in Abb. 1 ; Verwendung standardisierter Symbole gemäß DIN EN ISO 15223–1:2021. Reproduziert mit Genehmigung des DIN – Deutsches Institut für Normung e. V., Berlin.

**Abb. 4:** Erläuterung der Symbole aus Abb. 3 ; Verwendung standardisierter Symbole gemäß DIN EN ISO 15223–1:2021. Reproduziert mit Genehmigung des DIN – Deutsches Institut für Normung e. V., Berlin.

Chirurgische Instrumente sind analog zu behandeln: Auch hier sieht der Gesetzgeber vor, dass die Instrumente direkt auf dem Produkt (bspw. durch eine Lasersignierung) gekennzeichnet werden müssen. Das Symbol für die Ausweisung von CMR-Stoffen ist dabei ebenso verpflichtend.

## Konsequenzen der CMR-Kennzeichnungspflicht für die Hersteller von Medizinprodukten in Orthopädie und Unfallchirurgie

Die European Chemical Agency (ECHA) fasst auf ihrer Homepage sämtliche chemischen Stoffe zusammen, die sowohl die Anforderungen der MDR als auch der CLP- und der REACH-Verordnung erfüllen 11 .

Aktuell sind zum Stichtag 06.03.2024 insgesamt 6959 Stoffe gelistet, wovon bereits insgesamt 2592 potenziell unter die Rechtfertigungs- und Kennzeichnungspflicht für die Medizintechnikhersteller fallen (Überschneidungen durch die Eingruppierungen und Klassifizierungen sind hierbei möglich):

- 353 karzinogen 1A
- 856 karzinogen 1B
- 433 mutagen 1B
- 655 reproduktionstoxisch 1A
- 295 reproduktionstoxisch 1B

Diese Auflistung wird alle 3–6 Monate durch die ECHA aktualisiert. Nach einer Aktualisierung sind die MedTech-Hersteller mit einer Übergangszeit von 18 Monaten verpflichtet, die Kennzeichnungen zu erweitern, sofern ihre jeweiligen Produkte betroffen sind. Es ist zu erwarten, dass Hersteller in Bezug auf kennzeichnungspflichtige Stoffe mit einer sehr starken Dynamik konfrontiert sein werden.

Implantatwerkstoffe der großen Gelenke sind über die letzten Jahrzehnte materialseitig nicht gravierend verändert worden, sodass der Aktualisierungsaufwand dem Grunde nach beherrschbar bleibt. Aufgrund der MDR-bedingten Neuzertifizierung aller Bestandsprodukte müssen jedoch auch unveränderte und langjährig etablierte und erfolgreiche Produkte durch die neuen Kennzeichnungspflichten überarbeitet werden.

Es wird den Patienten schwer zu vermitteln sein, dass eine CMR-Kennzeichnung eines implantierten Produktes nicht zwangsläufig auch eine Gefährdung darstellt.

Zusätzlich zum verpflichtenden Implantatausweis und den bisherigen Informationen erhalten Patienten zukünftig auch Hinweise über das Vorhandensein von CMR-Stoffen. Inwieweit Patienten in die Lage versetzt werden können, diese Hinweise ohne Vorkenntnisse adäquat zu bewerten oder ob diese Informationspflicht im Gegenteil implantatbezogen zu erheblichem Vorbehalt, Ablehnung oder Verängstigung führt, muss genau beobachtet werden. Weiterführende Produktinformationen wie bspw. die Gebrauchsanweisungen liegen nämlich Patienten i. d. R. nicht vor.

**Zusatzinfo**

Diese Kennzeichnungspflicht besteht grundsätzlich, und zwar unabhängig davon, ob eine konkrete Gefährdung von dem (Implantat-)Werkstoff für Patienten ausgeht oder nicht.

## Konsequenzen der CMR-Kennzeichnungspflicht aus medizinischer Sicht und die aktuelle und zukünftige Versorgung

Aus medizinischer Sicht sollte jeder endoprothetisch versorgende Arzt über die Gründe für die CMR-Kennzeichnung, die wissenschaftlichen Grundlagen und die regulatorischen Anforderungen informiert sein. Dies gilt generell beim Einsatz von CMR-gekennzeichneten Implantaten, ganz besonders aber bei Implantaten, bei denen es konstruktiv bedingt zu einem Metallabrieb kommen kann.

Metall-Metall-Gleitpaarungen (MoM) fanden in den frühen 2000er-Jahren eine breite Verbreitung, sind jedoch heute nur noch sehr selten im Einsatz. Der aktuelle Anteil von MoM-Gleitpaarungen in der Hüftgelenkendoprothetik liegt in Deutschland bei ca. 0,1% 12 . Grund für den heute seltenen Einsatz der Gleitpaarung ist die beobachtete erhöhte Freisetzung von Metallionen durch Abrieb oder Korrosion in ungünstigen Situationen 13 . Derzeit gibt es keine gesicherten Hinweise darauf, dass die Implantate karzinogene oder teratogene Wirkungen haben. Allerdings sind die vorhandenen Daten nicht ausreichend, um solche möglichen Wirkungen vollständig auszuschließen. Im Urin oder Blut kann die Metallionenkonzentration bestimmt und erhöhte Konzentration nachgewiesen werden, die Hinweise auf eine lokale oder systemische Metallose geben können 14 . Allerdings gibt es keine verbindlichen Grenzwerte 15 .

So stellt ein bekanntes Problem der Pseudotumor dar, der bei ca. 1% der Patienten innerhalb der ersten 5 Jahre aufritt 16 . Zusätzlich stellt in der endoprothetischen Versorgung eine mögliche Kontaktallergie auf Metalle ein Problem dar. Diese wird für Nickel mit 20,4%, bei Kobalt mit 3,4% und für Chrom mit 1,5% in angegeben 17 . Für diese Fälle werden hypoallergene Prothesenvarianten verwendet, die auf unterschiedliche Art beschichtet (Titan-Niob-Nitrid) werden können. Im Vergleich zu Standardprothesen konnte in der primären Knieendoprothetik für die Kobaltkonzentration im Blutplasma im 5-Jahres-Vergleich trotz des technischen Vorteils jedoch kein signifikanter Unterschied festgestellt werden 18 . Da Allergiepatienten häufig unter Angststörungen leiden, besteht das Risiko durch die Kennzeichnung von Gefahrenstoffen, das Outcome weiter zu verschlechtern 19 .

Vorsorglich veröffentlichten EFORT (European Federation of National Associations of Orthopaedics and Traumatology) und die Orthopädische Sektorgruppe von MedTech Europe am 14. August 2018 eine gemeinsame Erklärung. In dieser wurde betont, dass die Kennzeichnungsvorschrift, unabhängig davon, ob eine CMR-Substanz ein tatsächliches Risiko darstellt, die medizinische Gemeinschaft möglicherweise in die Irre führen könnte. Die Empfehlung war daher, dass klargestellt werden sollte, dass zwar die Substanz selbst als gefährlich eingestuft wird, jedoch alle Risiken, die sich aus ihrem Vorhandensein ergeben, ordnungsgemäß bewertet, minimiert und bewältigt wurden 20 . Darüber hinaus veröffentlichte der Verband der Deutschen Dental-Industrie e. V. (VDDI) ein Statement zu Kobalt in Dentallegierungen mit dem Ergebnis, dass Kobalt-Basis-Legierungen weiterhin ein wertvolles und derzeitig nicht zu ersetzendes Therapiemittel in der Zahnheilkunde darstellen 21 . In der Tat kann die CMR-Kennzeichnung zu allgemeiner Verunsicherung führen, da sie bisher nicht standardmäßig in Aufklärungsgesprächen genannt wird und Patienten bspw. erst durch die Kennzeichnung in ihrem Implantatausweis ( Abb. 2 ) darauf aufmerksam werden könnten. Dabei ist es selbst bei dem überwiegenden Anteil an Patienten mit implantierten MoM-Gleitpaarungen unwahrscheinlich, dass es kurz- bis mittelfristig zu assoziierten Komplikationen aufgrund ihrer anteiligen CMR-Stoffe kommt 22 .

Um unbegründete Ängste von Patienten zu vermeiden, sind aus medizinischer Sicht der transparente und informative Umgang mit dieser Thematik sehr zu empfehlen. Dies könnte in Form von herstellerseitig erstellten Informationsmaterialien in digitaler und/oder Print-Version geleistet werden, sodass die Patienten in leicht verständlicher Form über die CMR-Kennzeichnung und ihre Implikationen informiert werden.

## Konsequenzen der CMR-Kennzeichnungspflicht aus ingenieurwissenschaftlicher Sicht und die zukünftige Versorgung

Die neuen Kennzeichnungsvorschriften sollen den Anwender darüber informieren, dass ein bestimmtes Medizinprodukt (z. B. ein Implantat) einen CMR-Stoff enthält, sind aber auch als Bestätigung dafür zu verstehen, dass der Hersteller alle von diesem Stoff ausgehenden Risiken beherrscht. Daher ist die Kennzeichnung an sich kein Anlass zu Bedenken hinsichtlich der Sicherheit von orthopädischen Implantaten.

Die Konzentration des CMR-Stoffes, der in der Legierung des Implantates enthalten ist, spielt beim Patienten die ausschlaggebende Rolle für das Auftreten von Problemen. Nur bei massiven Implantatproblemen kann die Konzentration so ansteigen, dass eine gesundheitliche Gefährdung für den Patienten besteht. Diese Thematik wurde durch die Einführung der Metall-Großkopf-Hüftgelenkersatzprothesen massiv befeuert, da für diese Prothesenart kritisch hohe Kobalt- und Chromkonzentrationen mit klinischen Konsequenzen beim Patienten häufiger auftraten. Ohne diesen Prothesentyp und ohne Patienten, die nach einer Keramikkopffraktur mit einem Metallkopf versorgt wurden 23 24 , ist das Risiko der Sterblichkeit, Herz-Kreislauf-Erkrankungen, Krebs und neurodegenerativen Erkrankungen für Patienten mit Kobalt und Chrom enthaltenden Hüftprothesen nicht erhöht 25 .

Bei der Knieendoprothetik ist Metallabrieb eine äußerst seltene Ursache für das Versagen eines totalen Knieersatzes und wurde eigentlich nur berichtet, wenn es zu einem anormalen Metall-auf-Metall-Kontakt oder massivem Drittkörperverschleiß gekommen ist 26 . Dafür spielt beim Kniegelenkersatz die Allergiethematik eine häufig diskutierte Rolle, wobei hier die wissenschaftliche Bewertung sehr heterogen ist und kein direkter Bezug zur CMR-Thematik besteht.

Als Beispiel wird vertieft auf die Kobaltthematik eingegangen. Eine Bewertung der wissenschaftlichen Literatur ergibt eindeutig, dass zwar eine potenzielle Gefährdung besteht, dass Kobaltlegierungen aber aufgrund ihrer einzigartigen Kombination von Eigenschaften wie Festigkeit, Haltbarkeit und einer langen Geschichte der sicheren Verwendung, sie für den Einsatz in einer Vielzahl von Medizinprodukten besonders geeignet machen 27 .

Die Bewertungen der relevanten präklinischen und klinischen Daten zur Karzinogenität und Reproduktionstoxizität von Kobaltlegierungen, die durchgeführt wurden, um den Anforderungen der EU-Medizinprodukteverordnung zu genügen, stützen die Schlussfolgerung, dass die Exposition gegenüber Kobaltlegierungen in Medizinprodukten über klinisch relevante Wege keine Gefahr für die Karzinogenität oder Reproduktionstoxizität darstellt 27 28 29 . Darüber hinaus ist das Risiko für unerwünschte Wirkungen, die bekanntermaßen bei erhöhten Kobaltkonzentrationen auftreten können (z. B. Kardiomyopathie) sehr gering (seltene Berichte, die oft ein einziges katastrophales Versagensereignis unter Millionen von Patienten widerspiegeln) 24 .

Zusammenfassend lässt sich sagen, dass das günstige Nutzen-Risiko-Profil, auch im Vergleich zu möglichen alternativen Materialien, für die weitere Verwendung von Kobaltlegierungen in Medizinprodukten spricht.

Dass das Metall Kobalt durch die Europäische Kommission als krebserzeugend der Klasse 1B (vermutlich krebserzeugendes Potenzial) eingestuft wurde, stützte sich in erster Linie auf Daten aus Studien zur Inhalationskarzinogenität bei Nagetieren 23 .

Die Autorengruppe fertigte eine systematische Übersichtsarbeit und eine Metaanalyse an, um die Risiken für einzelne Krebsarten bei Kobaltexposition durch Gelenkersatz (TJR) oder berufliche Exposition (OC) zu bewerten. Die Ergebnisse wurden nach Expositionsart (OC oder TJR), Expositionsniveau (Metall-auf-Metall [MoM] oder Nicht-MoM), Nachbeobachtungsdauer (Latenzzeit: < 5, 5–10 oder > 10 Jahre) und Krebsinzidenz oder -mortalität (Bewertung der Nachweisverzerrung) stratifiziert. Aus 30 Studien (653104 Probanden, durchschnittliche Nachbeobachtungszeit von 14,5 Jahren) ging hervor, dass der Zusammenhang zwischen TJR/OC und Krebsrisiko für 22 von 27 Krebsarten gleich null, für 3 Krebsarten negativ und für Prostatakrebs und Myelom positiv war. Für viele Krebsarten wurden eine signifikante Heterogenität und große Schätzbereiche festgestellt. Es wurde kein signifikanter Anstieg der Schätzungen nach Expositionsniveau oder Nachbeobachtungsdauer beobachtet.

Die derzeitige Evidenz, einschl. der schwachen Assoziationen, der Heterogenität zwischen den Studien und keiner erhöhten Assoziation mit dem Expositionsniveau oder der Nachbeobachtungsdauer, reicht nicht aus, um zu dem Schluss zu kommen, dass für Personen, die bei TJR/OC Kobalt ausgesetzt sind, ein erhöhtes Risiko für die Entwicklung bestimmter Krebsarten besteht.

Zahlreiche Studien kamen zu einem ähnlichen Ergebnis. Zusammenfassend lässt sich sagen, dass Metaanalysen keinen Zusammenhang zwischen der Exposition gegenüber orthopädischen Implantaten, die Kobaltlegierungen oder Kobaltpartikel enthalten, im beruflichen Umfeld und dem Gesamtkrebsrisiko ergab, einschl. einer Analyse von Studien, die Metall-auf-Metall- mit Nicht-Metall-auf-Metall-Implantaten direkt verglichen 23 24 25 26 29 .

## Konsequenzen der CMR-Kennzeichnungspflicht aus juristischer Sicht

## Allgemeines

Eine medizinische Behandlung hat den allgemein anerkannten fachlichen Standards zum Zeitpunkt der Behandlung (sog. Facharztstandard) zu entsprechen, soweit nicht etwas anderes vereinbart wurde, vgl. § 630a Abs. 2 BGB. Die Pflicht richtet sich unmittelbar an den Vertragspartner des Behandlungsvertrages – bspw. den Praxisinhaber oder den Krankenhausträger – mittelbar an den behandelnden Arzt, der nicht Vertragspartner geworden ist 30 .

## Behandlungsfehler

Entspricht die Behandlung nicht den allgemein anerkannten fachlichen Standards, liegt eine Verletzung der Pflichten aus dem Behandlungsvertrag in Form eines Behandlungsfehlers vor. Dazu zählen auch die Verwendung eines fehlerhaften Medizinprodukts, die medizinisch nicht vertretbare Auswahl bzw. die fehlerhafte Anwendung eines Medizinprodukts oder die Verletzung sonstiger sicherheitsbezogener Organisationspflichten 31 .

Die CMR-Kennzeichnung beeinträchtigt weder die Verkehrsfähigkeit CE-gekennzeichneter Medizinprodukte noch indiziert sie deren Fehlerhaftigkeit. Dem Gesundheitsschutz der Patienten wird durch die erforderliche CE-Kennzeichnung 32 hinreichend Rechnung getragen, sodass die Verwendung CMR-gekennzeichneter Medizinprodukte per se kein Behandlungsfehler sein kann. Etwas anderes kann sich nur dann ergeben, wenn in der medizinischen Wissenschaft positive Kenntnisse über gesteigerte Gesundheitsgefahren infolge der Verwendung der CMR-gekennzeichneten Produkte bestehen.

## Aufklärung

Die Durchführung medizinischer Maßnahmen, insbesondere Eingriffe in den Körper oder die Gesundheit, bedürfen grundsätzlich der vorherigen Einwilligung des Patienten, vgl. § 630d Abs. 1 S. 1 BGB. Die Wirksamkeit der Einwilligung setzt eine entsprechende (sog. Risiko- oder Selbstbestimmungs-)Aufklärung voraus, § 630d Abs. 2 i. V. m. § 630e BGB.

Inhalt der Aufklärung sind u. a. die zu erwartenden Folgen und Risiken des Eingriffs, vgl. § 630e Abs. 1 S. 2 BGB. Daher muss der Patient auch über Risiken aufgeklärt werden, die sich infolge der Einbringung von Medizinprodukten ergeben 33 . Diese ist mündlich durchzuführen, § 630e Abs. 2 S. 1 Nr. 1 BGB, weshalb ein Verweis auf die Gebrauchsanweisung des Herstellers nicht genügt 31 .

Seit Längerem ist bspw. bei Prothesen mit Metall-Metall-Gleitpaarungen das Risiko des Metallabriebs bekannt, der u. a. zu Kobalt- und Chromeinlagerungen im menschlichen Körper führen kann 34 . Nach im Vordringen befindlicher Ansicht ist über diese Risiken aufzuklären 35 . Soweit sich die potenziellen gesundheitlichen Gefährdungen des Metallabriebs auf CMR-Eigenschaften der verwendeten Materialien zurückführen lassen, liegt ein Hinweis auf diese spezifischen Eigenschaften und ggf. damit verbundenen Risiken zumindest nahe.

Keiner Aufklärung bedarf es über rein theoretische Risiken, für die weder praktische Erfahrungswerte vorliegen noch diese in Untersuchungen bestätigt wurden 36 . Dies soll auch dann gelten, wenn die Gebrauchsanweisung Hinweise über theoretische, aber nicht aufklärungsbedürftige Risiken enthält 31 .

Vor diesem Hintergrund kann bspw. im Fall des Risikos von Metallabrieb bei Endoprothesen differenziert werden: Der Metallabrieb stellt kein theoretisches, sondern ebenso wie die damit einhergehenden und messbaren Metalleinlagerungen im menschlichen Organismus ein reales Risiko dar 37 , weshalb es einer entsprechenden Aufklärung bedarf. Dasselbe kann für die allgemein bekannten gesundheitsschädlichen Eigenschaften der entsprechenden Materialien, insbesondere von CMR-Stoffen angenommen werden. Etwas anderes kann hingegen für konkrete, infolge der Einlagerung von CMR-Stoffen befürchtete Gesundheitsbeeinträchtigungen gelten, soweit für diese bislang keine Erfahrungswerte in der medizinischen Fachwelt bekannt sind. In diesem Fall würde es sich um lediglich theoretische, mithin nicht aufklärungspflichtige Risiken handeln 38 .

Neben der Risiko- oder Selbstbestimmungsaufklärung ist gemäß § 630e Abs. 1 S. 3 BGB auch über Behandlungsalternativen aufzuklären. Dies ist der Fall, wenn mehrere medizinisch gleichermaßen indizierte und übliche Methoden zu wesentlich unterschiedlichen Belastungen, Risiken oder Heilungschancen führen können 39 . Nach Ansichten in der juristischen Literatur und Rechtsprechung ist die Auswahl hinsichtlich des Materials von Endoprothesen keine aufklärungsbedürftige Behandlungsalternative, solange vom ausgewählten Material nach medizinisch-fachlicher Erkenntnis zum Zeitpunkt der Behandlung keine erhöhten Gesundheitsgefahren ausgehen 40 .

## Folgen der CMR-Kennzeichnung von Medizinprodukten im behandlungsvertraglichen Kontext

Insoweit zeigt sich, dass die medizinprodukterechtliche Kennzeichnungspflicht von CMR-Wirkstoffen keine direkten Auswirkungen auf die behandlungsvertraglichen Pflichten hat. Soweit aufgrund von in den Implantaten enthaltenen CMR-Stoffen reale Gesundheitsrisiken für den Patienten – bspw. aufgrund eines wenn auch selten vorkommenden Metallabriebs – bestehen, ist der Patient hierüber im Rahmen der Risiko- und Selbstbestimmungsaufklärung zu informieren.

## Konsequenzen der CMR-Kennzeichnungspflicht aus Sicht der Patienten und Patientinnen – bereits implantierte Patienten und zukünftig zu implantierenden Patienten

Die neue CMR-Kennzeichnungspflicht für Medizinprodukte kann bei Patienten, sowohl bei denen mit bereits implantierten Prothesen als auch bei denen, die eine Implantation in Zukunft planen, zu Verunsicherung führen. Es besteht jedoch kein erhöhtes Risiko für bereits implantierte Produkte nur, weil sie nachträglich eine CMR-Kennzeichnung erhalten haben. Die CMR-Kennzeichnung reflektiert lediglich regulatorische Anforderungen und nicht eine Veränderung des Risikoprofils der bereits implantierten Produkte. Dass Patienten erst nachträglich von der CMR-Kennzeichnung ihrer Implantate erfahren, birgt jedoch die Gefahr unnötiger Verunsicherung und Angst vor Komplikationen. Diese Tatsache muss klar kommuniziert werden, um solche Ängste zu vermeiden und Vertrauen zu erhalten. So sollten Patienten darüber informiert werden, dass die Sicherheit und Effektivität ihrer Implantate unverändert bleiben.

Für die zukünftige Versorgung gilt ebenfalls, dass die Kennzeichnung generell zu keinem erhöhten Risiko führt. Die neuen Anforderungen bez. der Kennzeichnung bedeuten keinesfalls eine Verschlechterung der bestehenden Versorgung oder hinsichtlich der Qualität oder Sicherheit der Implantate. Die neuen Kennzeichnungsvorgaben bedeuten keine technischen Änderungen an den Produkten und somit keine Veränderung für die klinische Praxis und die Versorgungsqualität, die Kennzeichnung dient lediglich der Transparenz und dem Schutz der Patienten in den wenigen Fällen, in denen materialbezogene Probleme mit Implantaten auftreten.

Daher indiziert die CMR-Kennzeichnung keinen Behandlungsfehler bei der Verwendung entsprechender Implantate. Eine Aufklärungspflicht besteht weiterhin nur für solche Risiken, die im Zusammenhang mit der Verwendung eines Implantates in der medizinischen Wissenschaft nachweislich bekannt sind, wie bspw. im Fall von Metall-Metall-Gleitpaarungen unter Verwendung von Kobalt-Chrom-Legierungen. Sollten von einem Implantat bekanntermaßen erhöhte Gesundheitsgefahren ausgehen, empfiehlt sich eine Aufklärung des Patienten über mögliche Alternativen.

Alten und neuen Patienten sollte kommuniziert werden, dass die in der endoprothetischen Versorgung verwendeten Implantate seit Jahren erprobt und sicher sind.

## Zusammenfassende Empfehlungen – was kommt auf zu uns zu? Ausblick

Für Patienten werden sich durch die CMR-Kennzeichnungspflicht keinerlei Veränderungen in der Versorgungsqualität ergeben. Hingegen stellt sich für die Ärzteschaft die zusätzliche Aufgabe, ihren Patienten das Gefühl der Sicherheit trotz einer nicht zu kontrollierenden Diskussion in der Öffentlichkeit zu vermitteln. Hier kann die Verwendung eines Keramikkopfes anstelle eines Metallkopfes beim Hüftgelenkersatz als Beispiel angeführt werden.

Wie beschrieben, geht von CMR-gekennzeichneten Implantaten nur ein sehr geringes Risiko aufgrund des Materials für den Patienten aus, das nur in sehr seltenen Fällen beim Versagen der Versorgung auftritt. Perspektivisch müssen Hersteller jedoch das Ziel haben, mehr CMR-freie Materialien zu qualifizieren. Allerdings ist dies unter den Regulierungen der MDR mit hohen Anforderungen, insbesondere zur klinischen Wirksamkeit, ein kosten- und zeitintensives Unterfangen.

Die bewährten Implantatwerkstoffe haben sich auch bei schwierigen Wechselsituationen als klinisch erfolgreich gezeigt. Der vollumfängliche Umstieg auf ein anderes Material ohne die entsprechende klinische Erfahrung könnte möglicherweise zu Einbußen bei der Bauteilsicherheit führen.

Die Umsetzung der CMR-Kennzeichnungspflicht sollte sowohl durch die Ärzteschaft als auch durch die Hersteller intensiv verfolgt werden, um frühzeitig einer Verunsicherung der Patienten entgegenwirken zu können.
